# Timing of Caffeine Therapy and Neonatal Outcomes in Preterm Infants: A Retrospective Study

**DOI:** 10.1155/2016/9478204

**Published:** 2016-05-08

**Authors:** Ivan Hand, Nahla Zaghloul, Lily Barash, Rudolph Parris, Ulrika Aden, Hsiu-Ling Li

**Affiliations:** ^1^Department of Pediatrics/Division of Neonatology, Kings County Hospital, Brooklyn, NY, USA; ^2^Department of Pediatrics, SUNY-Downstate Medical Center, Brooklyn, NY, USA; ^3^Cohen Children's Hospital at New York-North Shore Health System, Manhasset, NY, USA; ^4^Department of Women's and Children's Health, Karolinska Institute, Sweden; ^5^Department of Physiology and Pharmacology, SUNY-Downstate Medical Center, Brooklyn, NY, USA; ^6^Department of Biology, Medgar Evers College, City University of New York, Brooklyn, NY, USA

## Abstract

*Background*. Caffeine is widely used to treat apnea of prematurity. Here, we evaluated the efficacy of early caffeine (1-2 DOL) in decreasing the incidence of adverse neonatal outcomes.* Methods*. A retrospective cohort was used to compare the neonatal morbidity of 150 preterm neonates with gestational age ≤29 weeks. Infants were divided into 3 groups based on the initiation timing of caffeine therapy; (1) early caffeine (1-2 DOL), (2) late caffeine (3–7 DOL), and (3) very late caffeine (≥8 DOL).* Results*. The neonatal outcomes of early caffeine were comparable with those of the late caffeine group. Moreover, when comparing the neonatal morbidity of the very late caffeine group with that of the early caffeine group, multivariable logistic regression analyses were performed. We found that the timing of caffeine did not influence the risk of BPD (OR, 0.393; CI, 0.126–1.223; *p* = 0.107), but birthweight did (OR, 0.996; CI, 0.993–0.999; *p* = 0.018) in these infants.* Conclusion*. Neonatal outcomes of preterm infants were comparable whether caffeine was administered early or late in the first 7 DOL. The risk of BPD in infants receiving caffeine after 8 DOL was irrespective of delayed treatment with caffeine. Our results clearly demonstrate the need for further studies before caffeine prophylaxis can be universally recommended.

## 1. Introduction

Apnea of prematurity (AOP), defined as the cessation of breathing for more than 20 seconds, is commonly seen in preterm infants. Caffeine and other methylxanthines have been used to treat AOP for the past 40 years [1, 2]. However, the effectiveness and safety of caffeine therapy were first investigated rigorously later on by the multicenter trial led by Erenberg and colleagues as well as the international trial on caffeine for apnea (CAP trial) [3, 4]. In the CAP trial, infants with a birth weight of 500 to 1250 g, whose clinicians considered treatment with caffeine during the first 10 days of age, were randomly assigned to receive caffeine or placebo treatment. This study suggested a beneficial effect of caffeine on the incidence of bronchopulmonary dysplasia [4] as well as improved neurodevelopmental outcomes at 21 months [5]. A subgroup analysis of this randomized trial has further suggested early treatment with caffeine (≤3 days of life, DOL) is associated with a reduction in ventilation times when compared with infants with late caffeine treatment (4–10 DOL) [6].

Several studies have recently shown that early use of caffeine in preterm neonates was associated with decreased rates of death, bronchopulmonary dysplasia (BPD), and symptomatic patent ductus arteriosus (PDA) in the absence of adverse outcomes [7–10]. These studies, although well conducted, may contain a bias in outcome measures, favoring the prognosis of preterm infants with early caffeine. Preterm infants who were given caffeine demonstrated a high likelihood of being weaned off mechanical ventilation [11]. Clinicians administered caffeine shortly before planned extubation to prevent apneic episodes. In contrast, preterm infants with late caffeine were often associated with prolonged mechanical ventilation and poor health and were remote from extubation. Thus, the efficacy of early caffeine in benefitting pulmonary and neurological outcomes remains uncertain. Our hypothesis was that earlier administration of caffeine is a marker of a healthier preterm infant who would exhibit better neonatal outcomes. To test this hypothesis, this study included (1) preterm infants with caffeine in 1-2 DOL in the early caffeine group, (2) infants receiving caffeine in 3–7 DOL in the late caffeine group, and (3) infants receiving caffeine after 1 week of life (≥8 DOL) in the very late caffeine group. Moreover, the study included infants with gestational age (GA) ≤ 29 weeks. Preterm infants with GA > 29 weeks were excluded from this study since these infants have lower chance to develop neonatal morbidity and therefore are more appropriate to receive caffeine only after developing apnea [11].

## 2. Methods

### 2.1. Patient Population

A retrospective study was conducted on infants admitted to and treated in the Level III NICU of Kings County Hospital with gestational ages of 23–29 weeks, in the period from January 2010 to December 2014. Infants with major congenital malformations and those who died prior to discharge were excluded from this study. Preterm infants were divided into three groups based on the initiation timing of caffeine: the early caffeine group (1-2 DOL), the late caffeine group (3–7 DOL), and the very late caffeine group (≥8 DOL).

Clinical outcomes of preterm infants were assessed from chart review. Infants were analyzed for gender, birthweight (BW), and GA, as well as the maternal history which included mode of delivery and birth history (single versus multiple birth) (Table 1). The short-term outcomes which were evaluated in this study are shown in Table 2, which includes duration of NICU stay, bronchopulmonary dysplasia (BPD), respiratory distress syndrome (RDS), retinopathy of prematurity (ROP), and necrotizing enterocolitis (NEC). BPD was defined as requiring supplemental O_2_ therapy and/or respiratory support at postmenstrual age of 36 weeks [8, 10]. Infants who were discharged before 36 weeks on room air were defined as having no BPD. ROP was diagnosed by a pediatric ophthalmologist as part of routine screening and ROP was defined according to the international classification [12–14]. NEC was defined using Bell criteria [15]. The diagnosis of RDS was based on the presence of tachypnea, retractions or nasal flaring, grunting, central cyanosis, and chest radiograph [16].

### 2.2. Statistical Analysis

Descriptive statistics including frequencies and counts were performed separately for the total sample. Crosstabs and Fisher's Exact Test (for cell sizes ≤ 5) were used for comparisons of categorical variables between early and late caffeine use. In the presence of multiple variables such as GA and BW, a multiple logistic regression was performed to assess the effects of caffeine timing on neonatal outcomes. All analyses were done using SPSS® v23 (IBM, WA).

## 3. Results

A retrospective study was performed on preterm infants admitted to the NICU of Kings County Hospital from January 1, 2011, to December 31, 2014. This study population consisted of predominantly African Americans and a small percentage of other racial backgrounds. There were a total of 150 infants ≤29 weeks gestational age who met inclusion criteria for this analysis. The participating infants were divided into 3 groups based on the initiation timing of caffeine therapy and their neonatal outcomes were compared. Of 150 preterm infants, 85 infants received early caffeine (1-2 DOL), 46 infants received late caffeine (3–7 DOL), and 19 infants who received caffeine after 1 week of life (≥8 DOL) were included in the very late caffeine group. We did not observe any statistical difference in the neonatal outcomes of infants between the early and late caffeine group (i.e., 1-2 DOL and 3–7 DOL) (Table 2(a)), suggesting that preterm infants exhibited comparable neonatal morbidity whether receiving caffeine early of late in the first week of life. However, when we compared the outcomes of early caffeine with that of the very late caffeine group (≥8 DOL), our analysis showed that this very late caffeine group had higher incidence of BPD (47.4 versus 27.1%, *p* = 0.047) and PDA requiring ligation (10.5 versus 4.7%, *p* = 0.04) (Table 2(b)). We therefore examined the initiation timing of caffeine therapy for each of these infants (footnotes, Table 2) and found that a high percentage of these infants received caffeine after 14 days of life (66.7% and 100%, resp.), associated with the fact that preterm infants in this group were significantly more immature (25.94 wks versus 26.34 wks, *p* = 0.01) and smaller (835 g versus 891 g, *p* = 0.009). A multiple logistic regression analysis was therefore performed to incorporate these variables. We found that the timing of caffeine treatment did not influence the risk of BPD (OR, 0.393; CI, 0.126–1.223; *p* = 0.107) but the BW did (OR, 0.996; CI, 0.993–0.999; *p* = 0.018) in these infants (Table 2(c), top). Moreover, the incidence of PDA requiring ligation was comparable between the early and the very late caffeine groups (OR, 0.473; CI, 0.075–2.967; *p* = 0.424) (Table 2(c), bottom).

As a comparison, we performed additional analyses using similar criteria as previous reports [7–10]. Of the 150 infants, 85 were in the early caffeine group (1-2 DOL) and 65 infants received caffeine later (≥3 DOL). In contrast to previous reports, we did not observe any statistical difference in the tested neonatal morbidities, including BPD, ROP, RDS, PDA, and NEC between the early caffeine (1-2 DOL) and late caffeine groups (≥3 DOL) (Table 3).

## 4. Discussion

This single center study had several unique features which were designed to reevaluate the benefits of early caffeine on neonatal outcomes compared to that of late caffeine therapy. First, similar to previous reports [7–10], this study included infants with caffeine in the first 2 DOL in the early caffeine group. The selection for the late caffeine group was more stringent. Preterm infants with caffeine at 3–7 DOL were included in the late caffeine group, whereas those receiving caffeine after 1 week of life (≥8 DOL) were included in the very late caffeine group. Our results demonstrated comparable neonatal outcomes whether caffeine treatment was administered early or late within the first 7 DOL. In contrast, the early caffeine group appeared to show a lower incidence of BPD and PDA ligation only when compared with those receiving caffeine very late (≥8 DOL). However, since the mean GA and BW of this very late caffeine group were significantly lower, we speculate that this difference was due to the immaturity of these infants. Using multiple logistic regression to control these variables, we found that the initiation timing of caffeine was not associated with the risk of BPD. Moreover, there was no statistical difference in the incidence of PDA requiring ligation between the early and the very late caffeine groups. This finding is consistent with current neonatal practice, in which most infants start receiving caffeine treatment when the clinician feels that they are able to be weaned from ventilator support. That is, the early caffeine group often included healthier and more mature infants, whereas the late caffeine group included infants who received caffeine very late due to prolonged ventilation and greater immaturity. Together, this study unveiled the importance in patient selection when comparing the effects of early and late caffeine.

Several reports [7, 8, 10] have previously suggested greater benefits of early caffeine (0–2 DOL) in preventing BPD, lowering the incidence of PDA requiring ligation, decreasing length of mechanical ventilation, and decreasing NICU length of stay compared to caffeine treatment beyond this period (i.e., all infants with caffeine ≥3 DOL). Using the same grouping method, the mean GA and BW of both groups in our study population were similar. However, we did not observe any significant difference in neonatal outcomes of preterm infants with early or late caffeine, including BPD, ROP, and PMV (Table 3). This discrepancy may be due to differences in patient selection as described above. Moreover, we acknowledge that the small sample size in our study may affect the statistical power to detect any difference using this grouping method. The definition of BPD may also contribute to this discrepancy. Here, we defined BPD using the same criteria as two multicenter studies described [8, 10], whereas the single center study by Patel et al. was based on a web-based BPD estimator to predict the probability of BPD [7]. Lastly, this study included a relatively homogenous racial composition (>95% African Americans), whereas other studies had a mixed population.

Together, this study applied more stringent criteria to compare the neonatal outcomes of preterm infants with respect to the timing of caffeine treatment. We divided infants into 3 groups based on the initiation timing of caffeine: early (<3 DOL), late (3–7 DOL), and very late (≥8 DOL). We found that administering caffeine to preterm infants whether early or late in the first 7 DOL was likely to have comparable effects on neonatal morbidity. Moreover, the risk of BPD in the very late group (≥8 DOL) was not associated with delayed treatment with caffeine. Our results clearly demonstrate the need for a randomized controlled trial of early versus late caffeine in infants below 29 weeks' gestational age before early caffeine prophylaxis can be universally recommended.

## Figures and Tables

**Table 1:** Characteristics of preterm infants in this study^1^.

Variables	Early caffeine (1-2 DOL)	Late caffeine (3–7 DOL)	Very late caffeine (≥8 DOL)	*p* value
*N*	85	46	19	
Gestational age, wk	26.34 ± 1.84	26.41 ± 1.80	25.94 ± 2.01	*p* = 0.15
Birth weight, g	891.78 ± 227.50	909.13 ± 243.92	835.61 ± 188.70	*p* = 0.1
Sex (female/male)	38/47	28/18	9/10	*p* = 0.27
	(44.7%, 55.3%)	(60.9%, 39.1%)	(47.4%, 52.6%)	
Birth history (multiple birth)	14 (16.5%)	8 (17.4%)	1 (5.3%)	*p* = 0.48
Vaginal delivery	46 (54.1%)	16 (34.8%)	8 (42.1%)	*p* = 0.41
Chorioamnionitis	11 (12.9%)	8 (17.4%)	1 (5.3%)	*p* = 0.86
Apgar, 1 min	5.41 ± 2.26	5.61 ± 2.10	5.11 ± 2.13	*p* = 0.58
Apgar, 5 min	7.29 ± 1.45	7.31 ± 1.58	7.42 ± 1.17	*p* = 1.0
Surfactant	67 (78.8%)	35 (76.1%)	15 (78.9%)	*p* = 0.77
Ethnicity African American and Hispanic Black	95%	95%	95%	

^1^This study includes preterm infants with GA = 23–29 weeks and BW < 1500 g.

DOL, days of life.

**(a):** 

	Early caffeine (1-2 DOL)	Late caffeine (3–7 DOL)	*p *value
*N*	85	46	
GA (wks)	26.34 ± 1.84	26.41 ± 1.80	*p* = 0.53
BW (g)	891.78 ± 227.50	909.13 ± 243.92	*p* = 0.69
Sex (female/male)	38/47 (44.7%, 55.3%)	28/18 (60.9%, 39.1%)	*p* = 0.10

*Short-term clinical assessment*			
Duration of NICU stay	79.79 ± 30.51	76.73 ± 25.43	*p* = 0.18
BPD	23 (27.1%)	14 (30.4%)	*p* = 0.69
ROP requiring surgery	7 (8.2%)	0	*p* = 0.10
RDS	65 (76.5%)	36 (78.3%)	*p* = 1.00
PDA	41 (48.2%)	25 (54.3%)	*p* = 0.58
PDA requiring ligation	4 (4.7%)	3 (6.5%)	*p* = 0.70
Necrotizing enterocolitis	8 (9.4%)	5 (20%)	*p* = 0.39
PVL	4 (4.7%)	0	*p* = 0.30

DOL, days of life; BPD, bronchopulmonary dysplasia; ROP, retinopathy of prematurity; RDS, respiratory distress syndrome; PDA, patent ductus arteriosus; PVL, periventricular leukomalacia.

**(b):** 

	Early caffeine (1-2 DOL)	Very late caffeine (≥8 DOL)	*p* value
*N*	85	19	
GA (wks)	26.34 ± 1.84	25.94 ± 2.01	*p* = 0.01^*∗*^
BW (g)	891.78 ± 227.50	835.61 ± 188.70	*p* = 0.009^*∗*^
Sex (female/male)	38/47 (44.7%, 55.3%)	9/10 (47.4%, 52.6%)	*p* = 0.35

*Short-term clinical assessment*			
Duration of NICU stay	79.79 ± 30.51	77.13 ± 30.15	*p* = 0.25
BPD	23 (27.1%)	9 (47.4%)^1^	*p* = 0.047^*∗*^
ROP requiring surgery	7 (8.2%)	2 (10.5%)	*p* = 0.36
RDS	65 (76.5%)	15 (78.9%)	*p* = 0.74
PDA	41 (48.2%)	12 (63.2%)	*p* = 0.50
PDA requiring ligation	4 (4.7%)	2 (10.5%)^2^	*p* = 0.04^*∗*^
Necrotizing enterocolitis	8 (9.4%)	0	*p* = 0.1
PVL	4 (4.7%)	0	*p* = 1.0

(*∗*) means statistically significant.

^1^These infants received caffeine on days 8, 9, 9, 14, 15, 16, 22, 27, and 53.

^2^One infant received caffeine on day 19 and the other had caffeine on day 27.

DOL, days of life; BPD, bronchopulmonary dysplasia; ROP, retinopathy of prematurity; RDS, respiratory distress syndrome; PDA, patent ductus arteriosus; PVL, periventricular leukomalacia.

**(c):** 

		BPD		
	Odds ratio	95% confidence interval		*p* value
		Lower	Upper	

Initiation timing of caffeine therapy	0.393	0.126	1.223	0.107
GA (wks)	0.878	0.642	1.202	0.416
BW (g)	0.996	0.993	0.999	0.018^*∗*^

		PDA requiring ligation		
	Odds ratio	95% confidence interval		*p* value
		Lower	Upper	

Initiation timing of caffeine therapy	0.473	0.075	2.967	0.424
GA (wks)	0.842	0.467	1.518	0.567
BW (g)	0.997	0.991	1.004	0.404

(*∗*) means statistically significant.

DOL, days of life; BPD, bronchopulmonary dysplasia; PDA, patent ductus arteriosus; GA, gestational week; BW, birthweight.

**Table 3:** Neonatal outcomes of infants receiving early caffeine (1-2 DOL) and all infants with caffeine ≥3 DOL.

	Early caffeine (1-2 DOL)	Caffeine after day 3 (≥3 DOL)	*p* value
*N*	85	65	
GA (wks)	26.34 ± 1.84	26.28 ± 1.89	*p* = 0.35
BW (g)	891.78 ± 227.50	888.08 ± 239.72	*p* = 0.73
Sex (female/male)	38/47 (44.7%, 55.3%)	37/28 (56.9%, 43.1%)	*p* = 0.27

*Short-term clinical assessment*			
Duration of NICU stay	79.79 ± 30.51	75.72 ± 27.64	*p* = 0.47
BPD	23 (27.1%)	23 (35.1%)	*p* = 0.16
ROP requiring surgery	7 (8.2%)	2 (3.1%)	*p* = 0.10
RDS	65 (76.5%)	51 (78.5%)	*p* = 0.85
PDA	41 (48.2%)	37 (56.9%)	*p* = 0.61
PDA requiring ligation	4 (4.7%)	4 (6.2%)	*p* = 0.32
Necrotizing enterocolitis	8 (9.4%)	8 (12.3%)	*p* = 1.00
PVL	4 (4.7%)	2 (3.1%)	*p* = 0.27

DOL, days of life; BPD, bronchopulmonary dysplasia; ROP, retinopathy of prematurity; RDS, respiratory distress syndrome; PDA, patent ductus arteriosus; PVL, periventricular leukomalacia.
